# National survey of Hutchinson-Gilford progeria syndrome and progeroid laminopathy in Japan

**DOI:** 10.18632/aging.206277

**Published:** 2025-07-09

**Authors:** Yuko Okawa, Muneaki Matsuo, Rika Kosaki, Hidefumi Tonoki, Masanobu Fujimoto, Keiichi Ozono, Hiroyuki Saitou, Takuo Kubota, Yasuhisa Ohata, Noriyuki Namba, Shinjiro Akaboshi, Hirofumi Komaki, Natsuko Inagaki, Eiko Kato, Yoshihiro Maruo, Takahiro Yonekawa, Tomomi Nakamura, Katsuhiro Hayashi, Shinji Miwa, Miyuki Magota, Kenji Ihara

**Affiliations:** 1 Department of Pediatrics, Faculty of Medicine, Oita University, Yufu, Oita, Japan; 2 Department of Pediatrics, Faculty of Medicine, Saga University, Saga, Japan; 3 Division of Medical Genetics, National Center for Child Health and Development, Tokyo, Japan; 4 Department of Pediatrics, The University of Osaka Graduate School of Medicine, Suita, Osaka, Japan; 5 Medical Center for Translational Research Department of Medical Innovation, The University of Osaka Graduate School of Medicine, Suita, Osaka, Japan; 6 Center for Promoting Treatment of Intractable Diseases, Iseikai International General Hospital, Osaka, Japan; 7 Department of Pediatrics, Iseikai International General Hospital, Osaka, Japan; 8 Medical Genetics Center, Department of Pediatrics, Tenshi Hospital, Sapporo, Hokkaido, Japan; 9 Division of Pediatrics and Perinatology, Faculty of Medicine, Tottori University, Yonago, Tottori, Japan; 10 Department of Pediatrics, NHO Tottori Medical Center, Tottori, Japan; 11 Translational Medical Center, National Center of Neurology and Psychiatry, Kodaira, Tokyo, Japan; 12 Department of Clinical Genetics Center, Tokyo Medical University, Tokyo, Japan; 13 Department of Pediatrics, Tosei General Hospital, Aichi, Japan; 14 Department of Pediatrics, Shiga University of Medical Science, Otsu, Shiga, Japan; 15 Department of Pediatrics, Mie University, Tsu, Mie, Japan; 16 Department of Orthopaedic Surgery, Graduate School of Medical Sciences, Kanazawa University, Kanazawa, Ishikawa, Japan; 17 Department of Pediatrics, Yaeyama Hospital, Ishigaki, Okinawa, Japan

**Keywords:** Hutchinson-Gilford progeria syndrome, processing-deficient progeroid laminopathies, *ZMPSTE24* gene, *LMNA* gene, progerin

## Abstract

Background and Aim: Hutchinson-Gilford Progeria Syndrome (HGPS) and progeroid laminopathies (PL) are rare genetic disorders characterized by accelerated aging and early onset cardiovascular complications. Despite recent advances in the genetic diagnosis of HGPS and PL and the advent of lonafarnib treatment, the epidemiology and clinical characteristics of these disorders in Asia remain unclear. This study aimed to assess the prevalence, clinical features, and diagnostic trends of the HGPS and PL in Japan.

Methods: A nationwide two-step survey was conducted between July 2022 and January 2024, across 1,513 medical facilities.

Results: The survey identified ten HGPS patients, including eight with a confirmed genetic diagnosis. Early onset features such as scleroderma-like skin changes, growth retardation, and joint contracture were important in facilitating an early and accurate diagnosis. Cardiovascular complications typically occurred during their teens, and abnormalities in lipid metabolism were frequently observed. Overlapping but distinct phenotypes have been noted in *ZMPSTE24* deficiency and other laminopathies caused by *LMNA* pathogenic variants, such as Emery-Dreifuss muscular dystrophy. Four patients with definite HGPS and eight patients with progerin-related PL (definite HGPS, *n* = 4; uncertain HGPS, *n* = 2; *ZMPSTE24* deficiency, *n* = 2) were reported alive on October 2023, and the prevalence of HGPS was estimated to be 1 in 15.5 to 31.1 million.

Conclusions: This study provides updated epidemiological and clinical insights into HGPS and related laminopathies in Japan. The introduction of lonafarnib has the potential to extend survival, emphasizing the need to monitor for late-stage complications.

## INTRODUCTION

Hutchinson-Gilford progeria syndrome (HGPS, OMIM #176670) is an extremely rare genetic disorder first described by Jonathan Hutchinson in 1886 and Hasting Gilford, 1897 [1]. HGPS is one of the most severe genetic progeroid syndromes and is characterized by significant growth retardation, scleroderma-like skin, subcutaneous fat loss, alopecia, baldness, micrognathia, aged facial appearance, and joint contracture, which typically start to appear around 2 years of age [2]. Despite these characteristic physical manifestations, psychomotor functions and intelligence persist. The average life expectancy is reported to be 14.6 years with lethal complications due to atherosclerosis driven by dysfunction and apoptosis of vascular smooth muscle cells and endothelial cells [1, 3–6].

HGPS is caused by specific pathogenic variants of the *LMNA* gene, which encodes lamin A, a structural protein that provides physical support to the nuclear membrane [7]. Lamin A contributes to nuclear stability, chromatin organization, and functional interactions with transcription factors [8]. The pathological hallmark of HGPS is chronic cellular and tissue damage caused by “progerin,” which is an aberrant Lamin A protein produced by *LMNA* pathogenic variants. Laminopathies are characteristic diseases arising from lamin abnormalities and are classified into progerin-producing and non-progerin-producing types [9]. The former includes HGPS caused by *LMNA* pathogenic variants and distinct genetic disorders caused by *ZMPSTE24* pathogenic variants [10]. A classical-type *de novo* pathogenic variant (c.1824C>T; p.Gly608Gly) in exon 11 of the *LMNA* gene activates a cryptic splice site and causes aberrant splicing leading to the deletion of 50 amino acids near the C-terminus of prelamin A, including the endopeptidase-recognition sequence, producing “progerin.” Unlike normal lamin A, progerin retains its farnesyl group at the C terminal causing it to anchor permanently to the nuclear envelope. The accumulation of progerin disrupts nuclear architecture and impairs various cellular functions. Incorporation of progerin into the nuclear lamina leads to misshapen nuclei and the loss of peripheral heterochromatin. Cells expressing progerin exhibit defective responses to DNA damage and impaired repair mechanisms, contributing to genomic instability. Progerin also accelerates telomere shortening, promotes premature cellular senescence, and affects chromatin organization and gene transcription, leading to widespread transcriptional dysregulation [9, 11]. *ZMPSTE24* encodes a zinc metallo-endoprotease essential for the final maturation of lamin A [12]. During its synthesis, prelamin A undergoes farnesylation and carboxymethylation at the C-terminal CAAX motif, followed by the cleavage of its terminal 15 amino acids by *ZMPSTE24*. Functional loss of *ZMPSTE24* by biallelic pathogenic variants disrupts this cleavage process, leading to progeroid manifestations, such as mandibuloacral dysplasia type B or restrictive dermopathy type 1 [12, 13].

Progerin-related diseases have shown therapeutic responses to lonafarnib, a farnesyltransferase inhibitor that was approved by the U.S. FDA in November 2020 as the first medication for HGPS and processing-deficient progeroid laminopathies (PLs) [14]. A study by Gordon et al. demonstrated significant reductions in mortality over a 4-year period with Zokinvy® (lonafarnib), developed by Eiger BioPharmaceuticals [15, 16]. Following the approval of the drug in Europe, Australia, and China, it received regulatory approval in Japan in January 2024, with clinical application beginning in May 2024. The initiation of this treatment is expected to alter the clinical presentation, progression, and life expectancy of patients with HGPS. Improved survival may also reveal new pathological states and late-stage complications [15]. The prevalence rate and epidemiological data in Japan are influenced by enhanced diagnostic rates after recognition as treatable diseases.

Approximately 350–400 patients of classic HGPS have been reported worldwide. By December 2024, 151 classic HGPS patients were registered with the Progeria Research Foundation (PRF) in the U.S. As of September 30th, 2024, the prevalence of HGPS in the U.S. was estimated to be 1 in 20 million individuals (https://www.progeriaresearch.org/prf-by-the-numbers/). In comparison to Western countries, reports from Asia remain limited. In Japan, a primary survey of pediatric departments at 1,173 hospitals with over 200 inpatient beds was conducted approximately 10 years ago to investigate Japanese HGPS patients [17], and four Japanese patients were identified in a Japanese survey, and three Asian patients are included in the relevant literature. The clinical features of Asian patients, including sclerotic skin, growth failure, scalp alopecia, and severe cardiovascular and cerebrovascular complications, were fundamentally similar to those of other ethnicities [17].

This study aimed to re-evaluate the epidemiology of HGPS in Japan to determine its prevalence and clinical characteristics in Japanese patients. Therapeutic advancements are expected to alter disease progression and extend life expectancy, potentially revealing new complications. This study compared classic HGPS patients with milder variants and related laminopathies to offer insights into the evolving clinical course of HGPS patients under therapeutic intervention.

## RESULTS

### Overview

**Figure 1:**
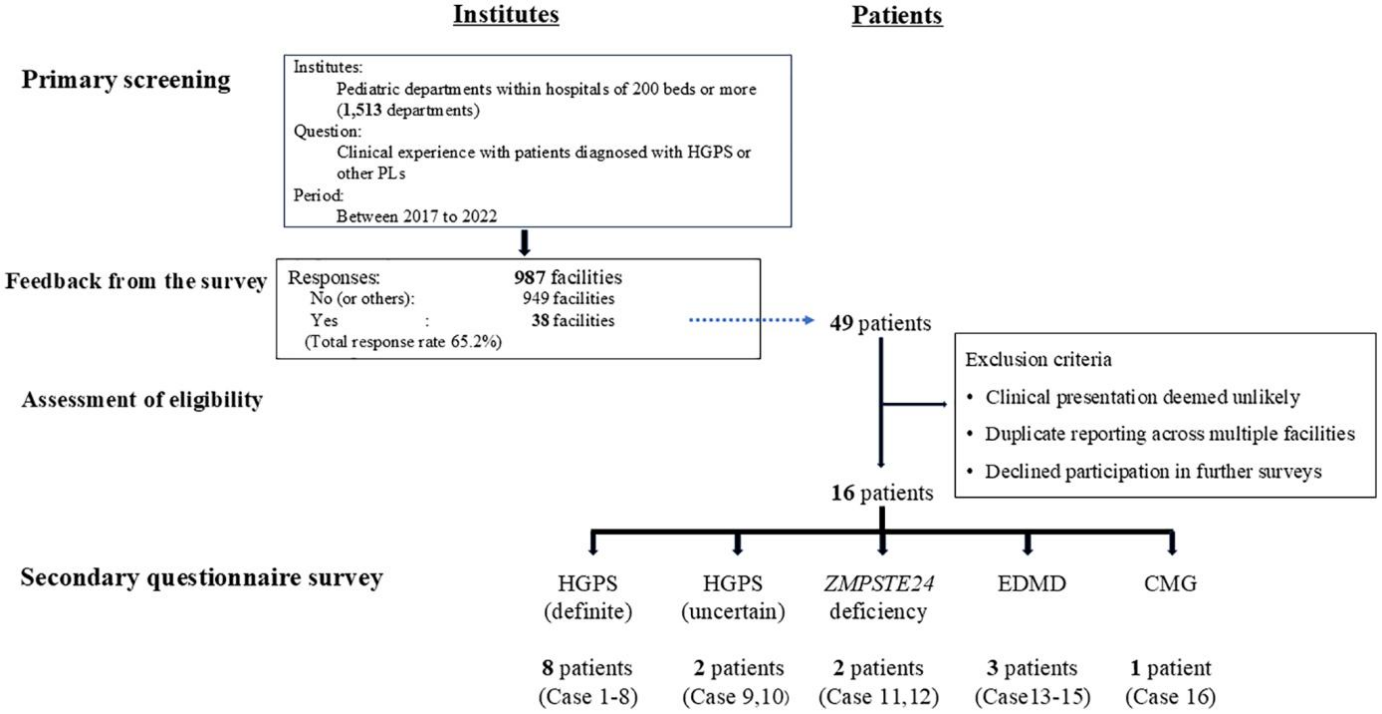
**Study flow diagram of the screening and following questionnaire survey for HGPS or laminopathy patients in Japan.** Abbreviations: PL: progeroid laminopathy; EDMD: Emery-Dreifuss muscular dystrophy; CMG: congenital muscular dystrophy.

Following the strategy of a previous nationwide survey conducted 10 years ago [17], primary surveys were distributed to 1,513 institutes and departments nationwide in Japan (Figure 1). Responses were received from 987 facilities (response rate: 65.2%). Among the respondents, 38 reported having diagnostic experience, with 28 facilities currently managing patients. From these responses, a total of 49 potential patients were initially identified, and 33 patients were excluded based on one or more of the following criteria: clinical presentations were deemed inconsistent or unlikely to represent HGPS or progeroid laminopathy, duplicate reporting from multiple institutions was detected, and the institutions or families declined to participate in further surveys. This screening process led to the selection of 16 patients for detailed evaluation through a secondary questionnaire survey.

The final cohort included the following diagnostic categories: eight patients (Cases 1–8) with HGPS (definite diagnosis); two patients (Cases 9 and 10) with HGPS (uncertain diagnosis); two patients (Cases 11 and 12) with *ZMPSTE24* deficiency; three patients (Cases 13–15) with Emery-Dreifuss muscular dystrophy (EDMD); and one patient (Case 16) with congenital muscular dystrophy (CMG) (Figure 1 and Supplementary Table 1).

### HGPS

#### Overview

A summary of the eight HGPS patients is presented in Supplementary Table 1. The patients consisted of four males and four females (Cases 1–8). Genetic diagnoses were confirmed in eight patients, including seven (Cases 1–7) with classic variants (c.1824C>T) and one (Case 8) with an atypical variant (c.1968+1G>A). Case 9 was a clinical diagnosis without a genetic analysis, and Case 10 was also clinically diagnosed with an *LMNA* variant of uncertain pathogenic significance (c.1762T>C; pCys588Arg). Therefore, the cohort was categorized into two groups: a confirmed group (Cases 1–8) and an unconfirmed group (Cases 9, 10).

In the confirmed group (definite HGPS), five of the eight patients were diagnosed before one year of age, while three patients were diagnosed before 6 months of age. In comparison, our previous survey, conducted 10 years ago, reported a median diagnosis age of 10 months for four Japanese patients, all of whom carried the classic c.1824C>T pathogenic variant. These findings suggest that accurate diagnoses were made before the formal establishment of diagnostic criteria in 2019 or the introduction of genetic testing under health insurance coverage in 2020, demonstrating the establishment of sufficient clinical and genetic diagnostic systems in Japan.

**Figure 2:**
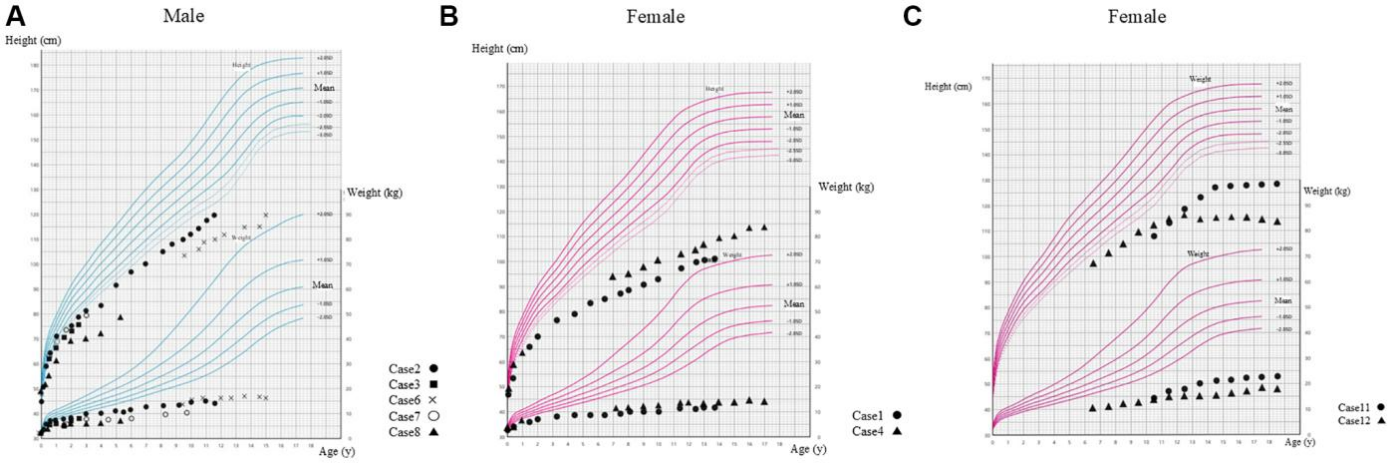
**Longitudinal growth chart for Japanese boys or girls.** (**A**) Male patients with HGPS; (**B**) Female patients with HGPS; (**C**) Female patients with *ZMPSTE24* deficiency.

Prenatal healthcare data and growth curves of the patients indicated that birth weights were within the standard range, and growth retardation was not evident during early infancy. However, deviations from the standard growth curves appeared significant from 1 year of age (Figure 2A, 2B). Similar growth trajectories were estimated in patients with *ZMPSTE24* deficiency (Figure 2C).

#### Clinical symptoms

**Figure 3:**
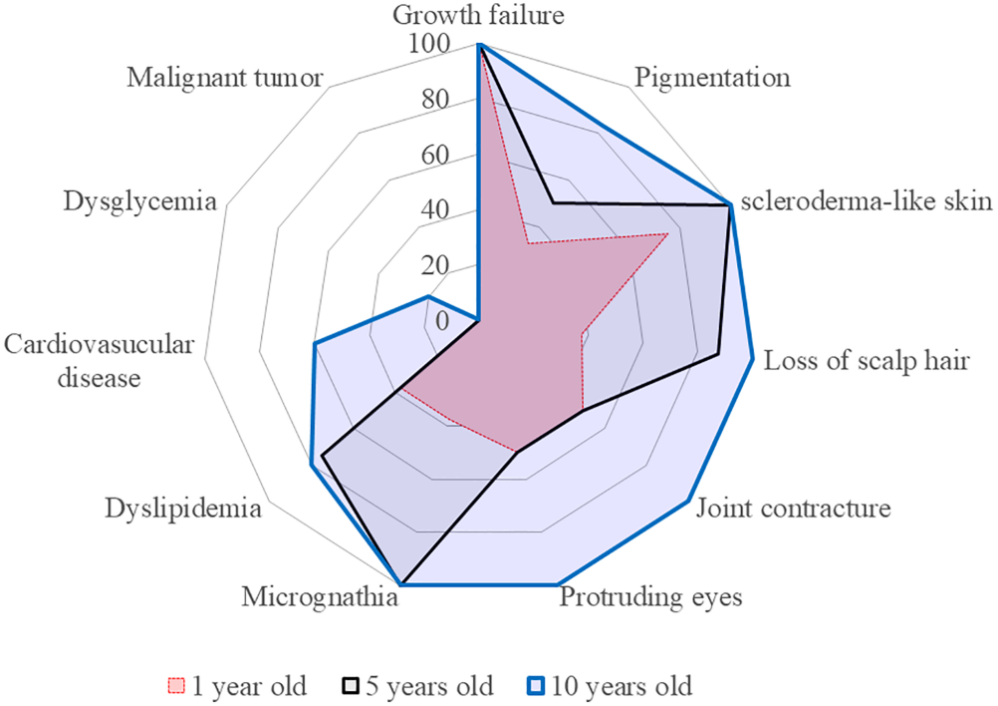
**The radar charts of characteristic symptoms and complications for definite HGPS (Cases 1–8).** Dyslipidemia: presence of high LDL cholesterolemia, low HDL cholesterolemia, or hypertriglyceridemia. Cardiovascular disease: hypertension, cerebral hemorrhage, cerebral infarction, angina pectoris, acute myocardial infarction, arteriosclerosis obliterans, or valvular heart disease. Dysglycemia: diagnosis of diabetes mellitus.

HGPS is characterized by progressive multisystem involvement resembling accelerated aging, including dermatological, skeletal, craniofacial, metabolic, and cardiovascular abnormalities. These symptoms generally become evident during infancy or early childhood, and intensify with age (Figure 3). All eight genetically confirmed patients demonstrated hallmark dermatologic changes, such as scleroderma-like skin thickening, alopecia, visibly dilated superficial veins, and subcutaneous fat atrophy. These findings typically emerge within the first year of life and remain stable thereafter. According to the radar chart, the dermatological manifestation of scleroderma-like skin thickening was observed in approximately 75% of patients at 1 year of age, with the prevalence reaching 100% prevalence at 5 years of age, supporting its value as an early diagnostic indicator.

External physical features, including joint contracture and loss of scalp hair, were consistently identified across age groups, and their high percentage in early childhood at 10 years of age on the radar chart suggests their utility in early clinical detection. Craniofacial characteristics, including micrognathia and prominent eyes, were observed with a high prevalence (100%) at 10 years of age. Lip cyanosis, a commonly described but variably recognized feature, was not clearly documented in 3 of the 8 patients (Supplementary Table 1), suggesting under-recognition rather than absence. Interestingly, cataracts were absent in the confirmed cases but noted in both unconfirmed cases (Cases 9 and 10), raising the possibility of late-onset or non-specific features. Pubertal development was poorly documented; however, menstruation was noted in one unconfirmed older female, indicating potential variability in gonadal involvement (Case 10). Notably, renal failure, not commonly described in HGPS, gradually appeared in one older patient, and was possibly related to longer survival (Case 9). Lipid metabolic disorders are the most common biochemical abnormalities. Six of the eight confirmed patients showed reduced HDL cholesterol levels, four had hypertriglyceridemia, and three had fatty liver disease (Supplementary Table 1). These abnormalities tend to appear progressively with age. The radar chart reflects this temporal pattern, with a low prevalence in younger patients and increasing percentages in adolescence, suggesting the age-related exacerbation of metabolic diseases.

**Figure 4:**
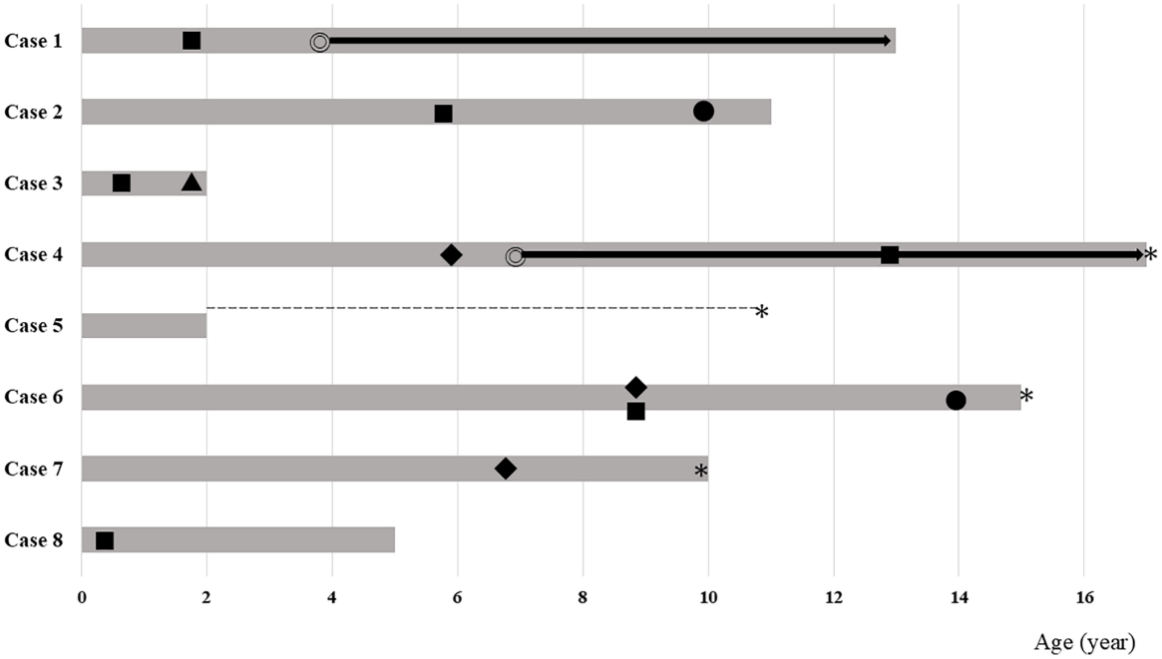
**Swimmer plots of definite HGPS. The period that was surveyed is indicated by a thick grey bar.** The periods with missing clinical data are indicated by dotted lines. For Case 5, detailed information was available up to 2 years of age, but further clinical information was not available, except for death due to arrhythmia at 11 years of age. ◎ Start of lonafarnib treatment, ● Occurrence of dysglycemia, ■ Occurrence of dyslipidemia, ◆ Cardiovascular event, ▲ starting GH treatment, * Fatal event (death).

Cardiovascular complications were infrequent in early childhood but became prominent before 10 years of age. Two patients of 11 and 24 years of age developed hypertension (Cases 6 and 9). Major ischemic events (e.g., myocardial infarction, stroke, and valvular heart disease) typically emerge during adolescence and are the primary causes of death. Several patients develop heart failure or arrhythmias, consistent with the known progression of atherosclerosis in HGPS. The swimmer plots (Figure 4) further illustrate the onset of metabolic or cardiovascular abnormalities and mortality, along with the timing of therapeutic interventions (e.g., growth hormone or lonafarnib). These data demonstrated a distinct age-related increase in cardiovascular event frequency, underscoring the need for early risk assessment and intervention.

#### HGPS epidemiology

##### Estimation of incidence

Birth and HGPS diagnoses over the past 20 years in Japan are summarized in Table 1. Two patients were diagnosed from 2014 to 2013, those were identified in the past survey, and two were newly diagnosed from 2013 to 2023. Three patients had the classic variant and one had a non-classic variant.

**Table 1:** Annual births and new HGPS patients in Japan.

**Year**	**Number of births**	**Number of new HGPS patients**
2004	11,10,721	0
2005	10,62,530	0
2006	10,92,674	0
2007	10,89,818	0
2008	10,91,156	0
2009	10,70,036	0
2010	10,71,305	1 (c.1824C>T)
2011	10,50,807	0
2012	10,29,817	1 (c.1824C>T)
2013	10,03,609	0
2014	10,03,609	0
2015	10,05,721	0
2016	9,77,242	0
2017	9,46,146	0
2018	9,18,400	1 (c.1968+1G>A)
2019	8,65,239	0
2020	8,40,835	0
2021	8,11,622	1 (c.1824C>T)
2022	7,70,747	0
2023	7,27,277	0

##### Estimation of prevalence

Four patients with definite HGPS and eight with progerin-related PL (definite HGPS, *n* = 4; uncertain HGPS, *n* = 2; and *ZMPSTE24* deficiency, *n* = 2) were reported alive in October 2023, and the estimated Japanese population was 124,352,000 in 2023. Hence, the HGPS prevalence was estimated to be 1 in 15.5 to 31.1 million.

### *ZMPSTE24* deficiency

#### Overview

*ZMPSTE24* deficiency involves the production of the abnormal protein progerin [12, 13]. Two siblings under management at the same facility were identified, aged 20 and 24 years, at the time of the survey. Diagnoses were made at 1 and 3 months of age. In the Japanese sisters, compound heterozygous mutations were detected in exon 1 (c.121C>T; p.Q41*) and exon 6 (c.743C>T; p.P248L) of the *ZMPSTE24* gene [18].

#### Clinical symptoms

Both siblings exhibited remarkable skin and skeletal abnormalities, as well as progeroid facial features (Supplementary Table 1, Cases 11 and 12). Osteoporosis was diagnosed at 9 and 3 years of age, respectively. Progeroid facial features and skin abnormalities appeared before the age of one in both patients, with full clinical presentation by age 10.

Their growth trajectory deviated from normal curves starting around one year of age, mirroring the HGPS phenotype (see Figure 2C). Lipid abnormalities appeared milder in comparison to HGPS, but individual differences were noted, as one sibling developed lipidemia after 16 years of age. These data suggest a similar age-related trend in the emergence of metabolic complications, although at lower prevalence rates in younger patients in comparison to patients with HGPS.

Despite the lack of intellectual impairment, both siblings received education at a special needs school because of physical disabilities. After graduation, they engaged in simple clerical or artistic work, such as patchwork and handicrafts, emphasizing the need for comprehensive social support despite a preserved cognitive function.

### Other laminopathies

#### Overview

Three patients of Emery-Dreifuss muscular dystrophy (EDMD) and 1 patient of congenital muscular dystrophy associated with *LMNA* pathogenic variants [19, 20] have been reported (Supplementary Table 1, Cases 13–16).

#### Clinical symptoms

Unlike HGPS, EDMD patients did not exhibit growth retardation or progeroid facial features during early infancy. However, joint symptoms, including contracture and scoliosis, were prominent and were observed as early as 6 years of age. Osteoporosis was observed during adolescence in all 3 EDMD patients, whereas metabolic abnormalities were not apparent. Severe cardiac conduction defects have been reported to be a significant complication.

### Disease comparison

A comparison of HGPS, *ZMPSTE24* deficiency, and other laminopathies is presented in Supplementary Table 1. HGPS and *ZMPSTE24* deficiency shared similarities in remarkable growth retardation and skeletal abnormalities, whereas EDMD exhibited juvenile-onset osteoporosis without significant growth retardation. Lipid metabolism markers may serve as therapeutic efficacy indicators for HGPS and *ZMPSTE24* deficiencies. Additionally, regular assessment of bone density and electrocardiography may help in the early detection of osteoporosis and arrhythmia.

## DISCUSSION

This study demonstrated the characteristic findings of patients with HGPS in Japan based on a report from a previous survey about 10 years ago [17]. The incidence has remained stable, with approximately 1 patient diagnosed almost every 5 years over the past two decades, reflecting the hereditary nature of HGPS caused by spontaneous pathogenic variants at specific sites in *LMNA* gene.

Data on the long-term growth curve have clarified the growth trajectory of Japanese HGPS patients, highlighting deviations beginning around 1 year of age. Cerebrovascular complications remain a major concern, but cardiovascular complications, such as vascular damage and arrhythmias, tend to emerge during school age and later in life. Kidney failure was reported as a cause of death in a long-term survivor in this study. Kidney damage appeared to occur in patients with Werner syndrome, a progeria syndrome with a relatively long-life span [21]. Therefore, the renal function should be carefully monitored as a potentially severe complication in patients with extended survival. Since lonafarnib is expected to improve life expectancy for patients with HGPS [15], the long-term complications of cardiovascular and renal diseases warrant close attention as late-stage complications [22, 23].

Comparisons of HGPS with *ZMPSTE24* deficiency revealed similarities in skeletal and skin phenotypes, with fewer cardiovascular and cerebrovascular events occurring in *ZMPSTE24* deficiency than in HGPS [24]. These differences may stem from variations in progerin production and its effect on vascular endothelial cells or hepatocytes, offering clinical insights for future basic research.

The worldwide prevalence of HGPS is consistent. According to PRF data of 154 HGPS patients worldwide, 18 were in the U.S., with an estimated prevalence of 1 in 19 million (https://www.progeriaresearch.org/prf-by-the-numbers/). In contrast, a Chinese study reported a median prevalence of 1 in 53 million across 17 provinces, with Hainan Province showing the highest prevalence at 1 in 23 million [25]. However, the centralized facilities in Beijing, China, achieved early and accurate diagnoses, with a median age at diagnosis of 3 months [26], emphasizing the importance of specialized multidisciplinary teams. The frequency of HGPS patients and the age at the diagnosis may reflect the universal healthcare system, accessible genetic testing, and a well-distributed network of specialized medical facilities spanning the entire country. This aligns with the prevalence of 1 in 15.5 or 31.1 million reported in Japan. These epidemiological data indicate that the prevalence in Japan is comparable to that in the U.S. and China.

These national surveys highlight that sex distribution, prevalence, incidence rates, and clinical features are largely consistent across Japan, the U.S., and China, suggesting little influence from racial or environmental factors. Disparities in the diagnostic age and prevalence likely reflect differences in awareness campaigns and the availability of multidisciplinary care based on the national health system. An early diagnosis and the initiation of standardized treatment are crucial for improving outcomes, particularly as Lonafarnib is currently approved for patients of ≥1 year of age.

In the future, clinical research should focus on identifying biomarkers to evaluate disease progression and treatment efficacy. Plasma progerin levels, for example, have been shown to decrease significantly with Lonafarnib therapy, suggesting a promising potential marker that has not yet been clinically applicable [27]. Comprehensive analyses of serum metabolites, combined with clinical and laboratory data, may provide deeper insights into the profiles of treated patients with HGPS [28].

This study had several limitations. First, the primary survey targeted only medical institutions with more than 200 beds, potentially excluding patients followed at smaller hospitals or clinics. Therefore, the prevalence of HGPS and related laminopathies may have been underestimated. Second, the response rate was 65.2% and not all eligible hospitals agreed to participate in the secondary survey, causing a selection bias and incomplete patient ascertainment. Third, while genetic testing was used to confirm the diagnoses in most patients with HGPS, some patients were categorized based on clinical features alone, potentially affecting diagnostic accuracy. Additionally, the heterogeneity of progeroid laminopathies, especially in non-classical forms such as *ZMPSTE24* deficiency and EDMD, limits the accurate estimation of the prevalence of these subtypes. Furthermore, detailed longitudinal data on lonafarnib treatment outcomes are unavailable. Finally, social, psychological, and quality of life aspects were not systematically evaluated, although they represent significant burdens for affected individuals and their families. Future prospective and multicenter studies are warranted to provide a more comprehensive understanding of these ultrarare conditions.

In conclusion, this study presents updated epidemiological data on HGPS in Japan, elucidates its clinical characteristics, and reports national prevalence estimates. Future international comparisons are important for understanding long-term outcomes and refining care strategies. Additionally, surveying the need for social resources to support patients throughout their lives remains a critical priority. Comprehensive research may provide further insights into the anticipated clinical course of patients with HGPS or progeroid laminopathies treated with lonafarnib.

## MATERIALS AND METHODS

### Primary survey

A national primary survey was conducted from July 2022 to March 2023 to evaluate the diagnostic experience with HGPS, suspected HGPS patients, and other PLs. The first questionnaire was delivered to 1,513 medical departments, including pediatric departments in facilities with at least 200 beds. The inclusion criteria were (1) hospitals with departments of pediatrics, neonatology, or genetics (individually or in combination) and at least 200 beds, (2) hospitals with genetics departments but without pediatrics or neonatology departments, and (3) specialized pediatric hospitals. Psychiatric and cancer hospitals were excluded from this study. The survey covered patients diagnosed or managed within the past five years from the time of the survey distribution. Facilities that had recently presented patient reports at academic conferences or had published studies were also included.

### Secondary survey

Based on the results of the primary survey, duplicate patients were identified and consolidated, and the diagnostic basis and accuracy were clarified through direct communication with attending physicians. In patients where a single patient visited multiple facilities or departments, the information was merged to avoid duplication. All the facilities that agreed to participate in the secondary survey were asked to complete a detailed survey. Ultimately, the study was conducted at 15 facilities.

### Reference to previous data

The analysis also included clinical data obtained from a national survey of our study group 10 years ago [17]. These historical data were incorporated into the estimation of prevalence.

## Supplementary Materials

Supplementary Table 1
