# Supplementary material for: National survey of Hutchinson-Gilford progeria syndrome and progeroid laminopathy in Japan
Source: Aging (Albany NY). 2025 Jul 9;17(7):1667–78. doi: 10.18632/aging.206277 (PMC12339027; doi:10.18632/aging.206277)
Supplement: Supplementary Table 1 [file aging-17-206277-s001.docx]

Table 1. Summary of the patients with laminopathy including HGPS (Hutchinson-Gilford progeria syndrome), unclassified laminopathy, mandibuloacral dysplasia,

EDMD (Emery-Dreifuss muscular dystrophy) and CMG (Congenital muscular dystrophies).

|  | 1 | 2 | 3 | 4 | 5 | 6 | 7 | 8 | 9 | 10 | 11 | 12 | 13 | 14 | 15 | 16 |
| --- | --- | --- | --- | --- | --- | --- | --- | --- | --- | --- | --- | --- | --- | --- | --- | --- |
| ■Diagnosis | HGPS | HGPS | HGPS | HGPS | HGPS | HGPS | HGPS | HGPS | Laminopathy (unclassified) | Laminopathy (unclassified) | mandibuloacral dysplasia type B | mandibuloacral dysplasia type B | EDMD | EDMD | EDMD | CMG |
| ■Sex | F | M | M | F | F | M | M | M | F | F | F | F | F | M | M | M |
| ■Genetic analysis |  |  |  |  |  |  |  |  |  |  |  |  |  |  |  |  |
| Gene | *LMNA* | *LMNA* | *LMNA* | *LMNA* | *LMNA* | *LMNA* | *LMNA* | *LMNA* | N/A | *LMNA* | *ZMPSTE24* | *ZMPSTE24* | *LMNA* | *LMNA* | *LMNA* | *LMNA* |
| Pathogenic variant | c.1824C>T | c.1824C>T | c.1824C>T | c.1824C>T | c.1824C>T | c.1824C>T | c.1824C>T | c.1968+1G>A | N/A | c.1762T>C | c.121C>T; p.Q41X (c.743C>T; p.P248L | c.121C>T; p.Q41X (c.743C>T; p.P248L | p.N456H | p.E358K | p.R41S | c.1147G>A  ; p.E383K |
| Age at diagnosis | 0y5m | 3y2m | 0y5m | 1y (clinical Dx）  5y (genetic Dx) | 2y | 2y7m | 0y8m | 0y4m | 5y | ND | 0y3m | 0y1m | 5y | 7y | 10y | ND |
| Age at investigation | 13y | 11y8m | 2y | 17y | 2y | 15y | 10y | 5y | 36y | 32y | 24y | 20y | 23y | 25y | 27y | 3y10m |
| Age at death | (Alive) | (Alive) | (Alive) | 17y | 11y | 15y | 10y | (Alive) | 36y | (Alive) | (Alive) | (Alive) | (Alive) | (Alive) | (Alive) | (Alive) |
| ■Growth |  |  |  |  |  |  |  |  |  |  |  |  |  |  |  |  |
| Gestational age | 37w | 38w3d | 38w6d | 38w0d | ND | 36w4d | 38w | 38w4d | 39w | ND | 37w5d | 37w0d | 40w | 40w | 40w | 41w2d |
| At Birth  Height  Weight | 47.0㎝  (-0.66SD)  2576g  (-1.06SD) | 45.0㎝  (-1.9SD)  2730g  (-0.68SD) | 46㎝  (-1.43SD)  2390g  (-1.53SD) | 49.0㎝(+0.29SD)  2838g (-0.4SD) | ND | 48㎝  (-0.48SD)  2720g  (-0.7SD) | 2580g  (-1.1SD) | 47.0㎝  (-0.95SD)  2342g  (-1.65SD) | 2800g  (-0.5SD) | ND | 40cm  (-4.0SD)  2230g  (-1.93SD) | 40cm  (-4.0SD)  2130g  (-2.18SD) | 2826g  (-0.44SD) | 43cm  (-2.86SD)  2500g  (-1.25SD) | 48cm  (-0.48SD)  2734g  (-0.67SD) | 50cm  (+0.48SD)  2968g  (-0.08SD) |
| At 1 month  Height  Weight  Weight gain | 51.6㎝  3360g  (+26.1g/day) | 51.0㎝  4030g  (+43.3g/day) | 50.5㎝  3125g  (+24.5g/day) | 52.7㎝  3666g  (+27.6g/day) | ND | ND | 51.0㎝  3415g  (+27.8g/day) | 50.5㎝  3752g  (+47g/day) | ND | ND | ND | ND | ND | ND | ND | ND |
| At 2 months  Height  Weight  Weight gain | ND | ND | ND  3995g  (+29g/day) | 57.0㎝  4305g  (+21.3g/day) | ND | ND | ND | 51.6㎝  4200g  (+14.9g/day) | ND | ND | ND | ND | ND | ND | ND | ND |
| At 4 months  Height  Weight  Weight gain | 54.4㎝  4800g  (+16g/day) | 58.9㎝  5800g  (+19.7g/day) | ND  4920g  (+15.4g/day) | 59.4㎝  4605g  (+5g/day) | ND | ND | ND | 56.3㎝  4740g  (+9g/day) | ND | ND | ND | ND | ND | ND | ND | ND |
| Last evaluation  Height  Weight | at 13y  101.4㎝  (-9.16SD)  11.5㎏  (-4.19SD) | at 11y  119.0㎝  (-3.51SD)  14.4㎏  (-2.69SD) | at 2y  76.5㎝  (-2.97SD)  7.7㎏  (-3.25SD) | at 17y  113.8㎝  (-8.48SD)、  12.8㎏  (-5.1SD) | at 11y  104.4㎝  (-5.87SD)  10.2㎏  (-3.46SD) | at 15y  120㎝  (-7.03SD)  16.3㎏  (-3.9SD) | at 10y  ND  10㎏  (-3.14SD) | at 5y  78.3㎝  (-6.6SD)  6.8㎏  (-4.36SD) | at 36y  134.4㎝  (-4.47SD)  23㎏  (-3.81SD) | ND | at 24y  113cm,  (-8.5SD)  17.6kg  (-4.49SD) | at 20y  113cm,  (-8.5SD)  17.5kg  (-4.5SD) | at 23y  145cm  (-2.47SD)  36.7kg  (-2.08SD) | ND | ND | at 3y10m  92cm  (-1.87SD)  8.8kg  (-3.42SD) |
| Growth curve | Fig.2 (B) | Fig.2 (A) | Fig.2 (A) | Fig.2 (B) | No | Fig.2 (A) | Fig.2 (A) | Fig.2 (A) | No | No | Fig.2 (C) | Fig.2 (C) | No | No | No | No |

(Continued)

|  | 1 | 2 | 3 | 4 | 5 | 6 | 7 | 8 | 9 | 10 | 11 | 12 | 13 | 14 | 15 | 16 |
| --- | --- | --- | --- | --- | --- | --- | --- | --- | --- | --- | --- | --- | --- | --- | --- | --- |
| ■Skin |  |  |  |  |  |  |  |  |  |  |  |  |  |  |  |  |
| Pigmentation  (age at onset) | No | Yes  (6y) | Yes  (1y) | ND | Yes  (1y) | Yes  (9y) | ND | Yes  (2y) | Yes  (0m) | No | Yes | Yes | No | No | No | No |
| Sclerodermatous  (age at onset) | Yes  (1m) | Yes  (3y) | Yes  (6m) | Yes  (2m) | Yes  (5m) | Yes  (0m) | Yes  (2m) | Yes  (2y) | Yes  (7m) | Yes  (8y) | Yes | Yes | No | No | No | No |
| Loss of scalp hair  (age at onset) | Yes  (14m) | Yes  (2y) | Yes  (1y) | Yes  (10m) | Yes  (1y) | Yes  (Infant) | Yes | Yes  (2y) | Yes | No | Yes | Yes | No | No | No | No |
| Prominent scalp veins  (age at onset) | Yes  (1y) | Yes  (6m) | Yes  (6m) | Yes  (10m) | Yes  (1y) | Yes  (Infant) | Yes | Yes  (2y) | Yes | No | Yes | Yes | No | No | No | No |
| Diminished subcutaneous fat  (age at onset) | Yes  (1m) | Yes  (5y) | Yes  (2m) | Yes  (10m) | Yes  (1y) | Yes  (10y) | Yes | Yes  (2y) | Yes | Yes  (8y) | Yes | Yes | No | No | No | No |
| Sagging skin  (age at onset) | Yes  (1m) | Yes  (6m) | No | Yes  (5y) | ND | Yes  (Infant) | ND | Yes  (2y) | Yes | No | Yes | Yes | No | No | No | Yes  (0m) |
| ■Skeletal system/joints |  |  |  |  |  |  |  |  |  |  |  |  |  |  |  |  |
| Delayed　closure of the anterior frontale  (age) | No | Yes  (6y) | Yes | ND | ND | ND | Yes | Yes  (5y) | ND | No | ND | ND | No | No | No | ND |
| Joint contracture  (age at onset) | Yes  (1m) | Yes  (6y) | ND | Yes  (2m) | ND | Yes  (9y) | Yes  (9y) | Yes  (0m) | Yes  (0m) | Yes  (teenager) | Yes  (0y9m) | Yes | Yes  (6y) | Yes  (6y) | Yes  (6y) | Yes  (0m) |
| Swelling of the distal finger  (age at onset) | ND | Yes  (8y) | ND | ND | ND | Yes  (9y) | ND | No | Yes | No | Yes | Yes | No | No | No | No |
| Finger melting statue  (age at onset) | ND | Yes  (3y) | Yes  (5m) | ND | ND | ND | ND | Yes  (4m) | No | Yes  (32y) | ND | ND | No | No | No | ND |
| Coxa valga  (age at onset) | Yes | ND | ND | ND | ND | Yes  (10y) | ND | Yes  (4y) | Yes  (18y) | No | Yes | Yes | No | No | No | No |
| Osteoporosis  (age at onset) | Yes  (7y) | ND | No | No | ND | ND | ND | ND | No | Yes  (30y) | Yes  (9y) | Yes  (3y) | Yes  (14y) | Yes  (15y) | Yes  (16y) | No |
| ■Face/Head |  |  |  |  |  |  |  |  |  |  |  |  |  |  |  |  |
| Aging appearance  (age at onset) | Yes | Yes  (7y) | Yes  (1y) | Yes  (1y) | Yes | Yes | Yes | Yes  (4y) | Yes | Yes  (10y) | Yes | Yes | No | No | No | No |
| Protruding eyes  (age at onset) | Yes | Yes  (7y) | Yes  (1y) | Yes  (1y) | Yes  (1y) | Yes  (9y) | Yes | Yes  (0m) | Yes | No | Yes | Yes | No | No | No | No |
| Micrognathia  (age at onset) | Yes | Yes  (7y) | No | Yes  (1y) | Yes  (1y) | Yes | Yes | Yes  (0m) | Yes  (0m) | Yes  (5y) | Yes | Yes | No | No | No | Yes |
| Cyanosis of the lips  (age at onset) | ND | No | Yes  (1y) | ND | Yes | Yes  (0m) | ND | Yes  (4y) | ND | Yes  (5y) | ND | ND | No | No | No | No |
| Cataract  (age at onset) | No | No | No | No | ND | No | ND | No | Yes  (6y) | Yes | No | No | No | No | No | ND |

(Continued)

|  | 1 | 2 | 3 | 4 | 5 | 6 | 7 | 8 | 9 | 10 | 11 | 12 | 13 | 14 | 15 | 16 |
| --- | --- | --- | --- | --- | --- | --- | --- | --- | --- | --- | --- | --- | --- | --- | --- | --- |
| ■Sexual maturity（female） |  |  |  |  |  |  |  |  |  |  |  |  |  |  |  |  |
| Puberty  (age at start) | No | Yes  (10y) | No | No | ND | ND | ND | No | ND | Yes | Yes | Yes  (11y) | Yes | Yes | Yes | ND |
| Menarche  (age at start) | No |  | No | No | ND |  |  |  | ND | Yes  (14y) | Yes | ND | Yes | No | No | No |
| ■Complications |  |  |  |  |  |  |  |  |  |  |  |  |  |  |  |  |
| １）Dysglycemia |  |  |  |  |  |  |  |  |  |  |  |  |  |  |  |  |
| Borderline dysglycemia | No |  | No | No | ND |  | ND | No | ND | No | No | No | No | No | No | ND |
| Diabetes  (age at onset) |  | Yes  (10y) | No | No | No | Yes  (14y) | ND | No | Yes  (22y) | No | No | No | No | No | No | ND |
| Drugs |  | Yes | No | No | No | Yes | ND | No | Yes | No | No | No | No | No | No | ND |
| ２）Dyslipidemia |  |  |  |  |  |  |  |  |  |  |  |  |  |  |  |  |
| High LDL cholesterolemia  (age at onset) | No | No | ND | No | ND | Yes  (9y) | No | Yes  (4y) | ND | No | Yes  (16y) | No | No | No | No | No |
| Low HDL cholesterolemia  (age at onset) | Yes  (2y) | Yes  (10y) | Yes  (1y) | Yes  (14y) | ND | Yes  (9y) | No | Yes  (11m) | Yes  (25y) | No | No | No | No | No | No | ND |
| Hypertriglyceridemia  (age at onset) | No | Yes  (6y) | No | Yes  (13y) | ND | Yes  (9y) | No | Yes  (4y) | Yes  (21y) | No | Yes  (18y) | No | No | No | No | No |
| Drugs | Yes | Yes | No | Yes | ND | Yes | No | Yes | Yes | No | Yes | No | No | No | No | No |
| ３）Fatty liver  (age at onset) | No | Yes  (8y) | No | No | Yes | Yes  (9y) | ND | No | No | No | No | No | No | No | No | ND |
| 4）Cardiovascular disease |  |  |  |  |  |  |  |  |  |  |  |  |  |  |  |  |
| Hypertension  (age at onset) | No | No | No | No | No | Yes  (11y) | ND | No | Yes  (24y) | No | No | No | No | No | No | No |
| Drugs | No | No | No | No | No | Yes | ND | No | Yes | No | Yes | No | No | No | No | ND |
| Cerebral hemorrhage  (age at the event) | No | No | No | No | No | Yes  (14y) | Yes  (10y) | No | No | No | No | No | No | No | No | No |
| Cerebral infarction  (age at the event) | No | No | No | Yes  (6y) | No | Yes  (9,10,11y) | Yes  (7y) | No | No | No | No | No | No | No | No | No |
| Angina pectoris/  　Acute MI  (age at the event) | No | No | No | AP　(9y)  AMI　(16y） | No | No | No | No | No | No | No | No | No | No | No | No |
| Arteriosclerosis obliterans  (age at the event) | No | No | No | No | No | Yes  (9y) | ND | No | Yes  (32y) | No | Yes  (20y) | No | No | No | No | ND |
| Valvular heart disease  (age at detection) | No | No | No | Yes | No | Yes  (15y) | No | No | No | No | No | No | No | No | No | ND |
| Valve |  |  |  | AR,AS |  | AS,MR |  |  |  |  |  |  |  |  |  |  |
| ５）Malignant tumor  (age at the onset) | No | No | No | No | No | No | No | No | Yes  Thyroid (18y) | No | No | No | No | No | No | ND |
| ■Cause of death | (Alive) | (Alive) | (Alive) | Heart failure | Arrhythmia | Heart failure | ND | (Alive) | Renal failure | (Alive) | (Alive) | (Alive) | (Alive) | (Alive) | (Alive) | (Alive) |
| ■Special notes |  |  | GH treatment |  | Hearing loss |  |  |  |  |  |  |  |  |  |  |  |
